# Employees and the National Insurance Act

**Published:** 1912-05-04

**Authors:** 


### NATIONAL INSURANCE ACT DEPARTMENT.

### EMPLOYEES AND THE NATIONAL INSURANCE ACT.

### IX.—The Contributions Payable—(*continued*).

We have already explained that the contributions of the employer and employee are not separate and distinct in the sense of being payable independently. In the first instance the employer has to pay both contributions, but he is given power to recover the amount of the employee's contribution, subject to certain restrictions. In the normal case, where wages are paid, the means of recovery is by deduction from such wage, and by that means only. The deduction may be made only for such period as is covered by the wages paid. Thus, if in one week the employer omits to make the deduction, when wages are payable weekly, he cannot rectify such omission in the following week or at any subsequent time. If wages are payable monthly the deduction will be four times the amount of the employee's weekly contribution, or, should there be five Mondays in the month for which the wages are paid, the authorised deduction will be five times the weekly contribution. The provision making it incumbent upon the employer to deduct the employee's contribution, if at all, from the wages paid for the period covered by such contributions, is a safeguard of the interests of the insured person. Not only does it relieve the employee of making the payment himself, which if left to himself he might sometimes forget or defer to a more convenient time, but it also insures that he shall not be embarrassed by more than the one deduction from any individual payment of wages. Further than this, any sum deducted by an employer is to be deemed to be entrusted to him for the purpose of paying the contribution in respect of which it was deducted, and any failure on the part of the employer to apply the money in this way will render him subject to penalties. Even if the insured person should acquiesce in the omission to pay the contributions it will be the employer who will suffer when the omission is detected.

#### Inducement to Become Insured.

As the benefits to be derived are not proportional to the contribution of the employed contributor, but to the joint contributions of himself, the employer, and the State, it will only be in rare cases that a person entitled to become insured will not desire to take advantage of a scheme so largely to his benefit. The compulsory character of the Act made it essential that there should be sufficient inducement to appeal to the self-interest of the insured person, and it is thought that such inducement has been amply provided by the relation of the benefits to the contributions—a relation which is commonly expressed as 9d. for 4d. It has been hinted fairly openly in certain quarters that there will be passive resisters to the Act. The employer who contravenes the provisions of the Act by failing to pay the contributions on behalf of his employee runs the risk of having to pay a fine, which may be as much as £10, in addition to having to make good the amount of the contributions he has failed or neglected to pay.

There are certain classes of persons to whom wages are not paid direct. Cab-drivers are an example. The owner lets the cab to the driver and receives payment from the driver for so doing, the latter retaining a proportion of his takings, or the balance of such takings, after paying a fixed sum to the owner, as his own remuneration. In cases of this description the employee's contribution cannot be recovered by means of deduction from the wage, and power is accordingly given to the employer to make the recovery as a civil debt, and in the event of it not being paid he can proceed by way of summons to obtain the return, provided action is taken within three months from the date when the contribution was payable. It is to be hoped that payment will be made by employees of this class without recourse to summary procedure.

#### Apprentices and Probationers.

Where the contributor is not paid wages or other money payments by his employer or any other person, the employer is liable to pay the contributions payable both by himself and the contributor, and is not entitled to recover any part thereof from the contributor. Thus it would seem that when an employer has an apprentice to whom he makes no money payments, but receives from him services, either without consideration or in return for board and lodging, the employer will have to pay the apprentice's contribution as well as his own. We have used the word apprentice, but this must not be taken as including a fully indentured apprentice, because the latter, when serving without money payments, is exempt from compulsory insurance. Probationers in hospitals and other like institutions, who are not serving under articles, and do not receive monetary wages, provided they are over sixteen years of age and are not exempted by any other provision of the Act, will have to be insured, and by reason of the fact that no wages are paid in money their contributions will have to be paid by the institution which employs them, and will not be able to be recovered from the probationers.

#### Casual Labour.

There are many employed persons who serve more than one master in the course of a week, and the question arises as to which of the employers must pay the contribution. The answer is furnished in section 5 of the Third Schedule, which provides that the first person employing such a person in that week, or such other employer or employers as may be prescribed by the Insurance Commissioners, shall be considered to be responsible for the payment of the contributions. No rules governing the case have as yet been issued by the Commission, but the Chancellor of the Exchequer has indicated in an official statement that arrangements will be made in connection with Labour Exchanges whereby the employers will be able to share the payment of the contributions amongst themselves, and thus relieve the first employer of the burden of the full weekly contribution. It is to be noted that the casual worker is only compulsorily insured when he is employed for the purpose of the employer's trade or business, and in consequence casual domestic workers do not fall within the scope of the Act.

### The Employer's Contribution.

The provisions with which we have already dealt refer to the employee's contributions, and indications have been given of the manner in which they may be recovered by the employer. We have seen that, with the exception of the case where no money wages are paid, the employer is entitled to recoup himself for the payments made on behalf of the insured person. Turning now to the employer's own contribution, it is laid down that, notwithstanding any contract to the contrary, the employer shall not be entitled to deduct from the wages of or otherwise to recover from the contributor the employer's contribution. This is as explicit as it well can be, and any attempt at infringement will be made the subject of heavy penalties. The insurance of the worker is presumed to be as much for the good of the employer as for the employed. More efficient work is to be the result of healthier workers. Seeing, then, that the employer is to benefit, even though such benefit may be indirect, it is argued that he should pay for it. Thus the necessity arose for preventing the imposition of the employer's contribution on the employee. There is nothing in the Act, however, to prevent an employer, if he can and wishes to do so, from terminating the engagements of his operatives by due notice and renewing them at wages reduced by the amount of the employer's contribution.

### Outworkers' Contributions.

The tenth section of the Third Schedule reserves to the Insurance Commissioners the power of framing regulations as to the contributions of outworkers. The idea of the section is to make allowance in the contribution for the amount of work done, instead of referring to the weeks in which it is done. This is a very difficult matter to solve, and the regulations will be awaited with interest. Difficulties beset the framers of the Act at every turn, and the problem of the outworker was one of the most intricate they had to face. Even now it is not clear that the provision allows any variation in the total contribution of employer and employee. If the benefits are to be granted at the standard rates, so also must the total contribution be at the rates prescribed by the Act. The regulations of the Commissioners would thus seem to be limited to the manner of collection of the contribution, and possibly also to a variation in the proportion of the contribution to be borne by the employer and employee respectively.

### Contributions for Uninsured Persons.

One of the fundamental principles underlying the Act is that the charge made upon the employer shall not be affected by the special provisions whereby persons of advanced age and those in receipt of pensions of at least £26 a year are exempt from insurance. An employer who contrived to obtain the services of exempted persons would have an advantage over his competitors who were not so fortunate in this respect, if it were not for the fact that the situation had been foreseen and provision made accordingly. The employer who obtains the services of those who, apart from their special exemption on the grounds indicated, would come within the description of employed contributors, still remains liable for his own contribution on their behalf, and such contributions will be carried to a special fund designed for the purpose. The disposition of the fund will be by regulations to be framed by the Insurance Commissioners.

### Voluntary Contributors.

At the commencement of the Act the class that will be known as voluntary contributors will not be employees in the usually accepted sense of that term. The class will be composed of those who are engaged in some regular occupation on their own account and are wholly or mainly dependent for their livelihood on the earnings derived by them from that occupation, such earnings not exceeding £160 per annum. In a further period of five years the voluntary class will be recruited from the ranks of the employed contributors, whose payments are made compulsorily. The compulsory insurance will cease when the income limit is reached, but the employed contributor who has been insured for at least five years will, in such circumstances, have the option of continuing as a voluntary contributor. At the outset the contributions of the voluntary contributor will depend upon his age on entering insurance. If he be under the age of forty-five on entering insurance, and such entry is made within six months from the commencement of the Act, the rate of contribution will be the same as that applicable to employed contributors. If he is over that age the rate will be according to a specially prepared scale depending on his age. The voluntary contributor, who has been insured for five years as an employed contributor, will continue his contributions at the employed rate. It must, however, be borne in mind that the voluntary contributor, of either description, will have to pay the whole contribution which in the case of employed contributors is borne proportionately by the employer and the employee, but the State provides its share of the benefits for him. The contributions of voluntary members cease to be payable on the attainment of the age of seventy in the same way as those of the employed contributor.

NOTE :—Questions and Correspondence on all doubtful points are invited, and should be addressed to the Insurance Editor, The Hospital, 28 Southampton Street, Strand, W.C.

